# Cutaneous Melanoma Management—An Update and Narrative Review for the General Surgeon

**DOI:** 10.1111/ans.70252

**Published:** 2025-09-12

**Authors:** Matthew Binks, Alexander C. J. van Akkooi, Alexander M. Menzies, Andrew Spillane

**Affiliations:** ^1^ Melanoma Institute of Australia Sydney New South Wales Australia; ^2^ Orange Health Service Orange New South Wales Australia; ^3^ Department of Melanoma and Surgical Oncology, Institute of Academic Surgery Royal Prince Alfred Hospital Sydney New South Wales Australia; ^4^ Faculty of Medicine and Health The University of Sydney Sydney New South Wales Australia; ^5^ Royal North Shore Hospital Sydney New South Wales Australia

**Keywords:** cutaneous melanoma, immunotherapy, surgery, surgical oncology

## Abstract

**Background and Aims:**

The multidisciplinary management of cutaneous melanoma is advancing rapidly. Surgeons remain a key entry point for melanoma patients into specialist care and their up‐to‐date knowledge is vital for patient outcomes.

**Methods:**

A narrative review was performed to provide an update for the surgeon working outside of a high‐volume melanoma centre.

**Results:**

This review covers recent developments in the management of both localised and metastatic cutaneous melanoma and how immunotherapy is changing the melanoma landscape.

**Conclusion:**

This review offers a review of the literature and perspectives on potential future developments in cutaneous melanoma management from the Melanoma Institute of Australia.

## Graphical abstract

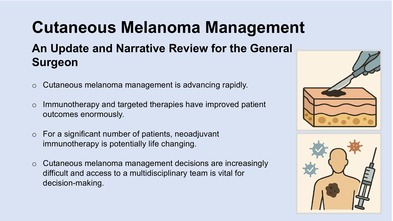

## 1 Introduction

Melanoma management is one of the most rapidly evolving fields of medicine in recent history. With surgical excision being the traditional mainstay of melanoma management, general, oncologic or plastic surgeons remain the first point of referral for many melanomas found in general practice, skin clinics or dermatology practice [1, 2]. Patients with intermediate risk and high‐risk melanoma have been recommended to have more accurate staging with sentinel node biopsy (SNB) for several decades now. In recent times, the recommendation for immediate completion lymph node dissection (CLND) for positive SNB has been altered as a result of two major international studies showing no melanoma‐specific survival (MSS) advantage with the procedure at the cost of a significant risk of morbidity, particularly lymphoedema [3, 4].

With recent advances in systemic treatments, such as immunotherapy agents and targeted therapies, the treatment paradigm for melanoma has changed significantly, with earlier incorporation of these effective systemic therapies into the standard treatment algorithm [5, 6, 7, 8, 9]. Patients that were heretofore treated with up‐front surgery, such as those with clinically detectable nodal metastases, are seeing marked improvements in event‐free survival (EFS) with neoadjuvant immunotherapy [10, 11]. Furthermore, patients with oligo‐progressive metastatic disease are now seeing benefits from metastasectomy. As such, surgeons the world over must be up to date with melanoma management to avoid the omission of lifesaving treatments.

The following review aims to provide an update on the management of cutaneous melanoma for the melanoma surgeon working outside of a high‐volume melanoma unit. It also offers perspectives on the trajectory of melanoma management and potential future developments from Melanoma Institute of Australia.

## 2 The Primary

Management of the primary site of melanoma involves the relatively straightforward process of a wide local excision (WLE). However, multiple factors require consideration before proceeding.

First, the clinical context is important. In the setting of melanoma, the surgeon is typically presented with an excision biopsy wound where a pigmented or evolving skin lesion once sat. The key questions in this setting are the certainty of the diagnosis, the clinicopathological stage of the disease, the subsequent surgical margins that are required and how to address the lymph nodal basin.

If a suspicious lesion is still in place, a decision regarding biopsy must be made. The well‐known ABCDE approach is best applied by assessing the lesion for asymmetry, an irregular border, varied colouring, a diameter > 6 mm and for evolution or change. Additionally, the lesion should be assessed for nodularity [12, 13]. Dermoscopy is a valuable tool in this assessment and can reduce the negative biopsy rate when performed by a trained clinician. A few hours training in the use of dermoscopy can significantly improve the ability to diagnose a melanoma [14]. Digital dermoscopy with the use of repeated assessment and artificial intelligence is emerging as a powerful diagnostic tool in dermatology clinics [15, 16, 17, 18].

Confocal microscopy has seen remarkable improvements in non‐invasive lesion assessment. The technique uses a handheld or wall‐mounted imaging microscope that magnifies views of the skin by 1000 times and produces images like that of a histopathological slide, but in a horizontal plane. Confocal microscopy is 92% sensitive for cutaneous melanoma [19, 20]. Confocal is particularly useful for mapping out the extent of in situ melanoma on the face or other areas where taking a wide margin will lead to increased morbidity [21]. The technique is highly specialised and the equipment is expensive and as such it is only available in major melanoma centres to date.

So as to avoid unnecessarily broad excisions of benign moles and to ensure accurate sentinel node biopsy in those that require it, biopsy of the primary should always precede wide excision [22, 23, 24]. An excision biopsy is ideal as it provides complete lesion information and if margins are negative or involved by in situ disease only, the urgency of the subsequent wide excision is reduced [25].

When confocal microscopy is unavailable for large in situ lesions or those in sensitive anatomical locations, such as facial lentigo maligna or acral lesions, partial biopsy is the pragmatic approach. This may involve an incisional biopsy or multiple mapping punch biopsies [26]. If a partial biopsy is opted for, note must be made that a single melanoma is often heterogeneous and partial biopsy risks under‐staging the lesion [25, 27]. Consequently, any partial biopsy should target the portion of the lesion likely to house the most advanced disease [28]. Dermoscopic guidance can be valuable, particularly in equivocal lesions, in which biopsies can be aimed at suspicious areas, such as blue‐white structures, which may indicate regression [29].

Communicating the history of the patient and lesion to the pathologist via the request form is vital and significantly improves diagnostic yield [30]. Histopathological uncertainty is not uncommon and sending the slides for a second opinion from a specialist melanoma pathology centre can improve diagnostic confidence and can result in upstaging or downstaging a lesion [31, 32, 33].

### 2.1 Margins of Excision

The surgical margin required to safely treat a melanoma has been the topic of debate for decades. Six randomised controlled trials examining margin de‐escalation were performed between 1980 and 2004 and none found a long‐term significant improvement in local recurrence or survival with wider margins compared to narrower [34, 35, 36, 37, 38, 39]. Current Australian and European guidelines recommend that melanoma in situ should be excised with a 5–10 mm margin, T1 disease with a 1 cm margin, T2a disease with a 1–2 cm margin and T2b disease and above with a 2 cm margin [40]. Desmoplastic melanoma requires a 2 cm margin.

The question of further margin de‐escalation is being examined by the MELMART II trial. An international, multicentre randomised controlled trial, the trial is randomising intermediate and thick melanomas (T2b and above) to either a 1 or 2 cm excision margin. The trial is soon to complete recruitment. Some have even suggested foregoing WLE altogether in case of clear margins on the excisional biopsy [41, 42, 43, 44, 45]. The Dutch NORMA, as well as the Swedish Watch‐and‐Wait (WOW) study are addressing this. Owing to its typically wide breadth and facial location, lentigo maligna can pose particular challenges when attempting to achieve negative margins [46, 47, 48]. Histopathological margin assessment is also difficult and 10 mm surgical margins or Moh's surgery are recommended. The topical immune response modifier, imiquimod, can be effective in challenging cases with the guidance of a specialist dermatologist [49]. MIA has a lentigo maligna clinic in which patients are discussed to determine the best management strategy between surgery, radiotherapy or imiquimod or combinations of these.

## 3 Detecting Microscopic Stage 3 Disease: *Deciding Who Should Have a Sentinel Lymph Node Biopsy (SNB)*

Since publication of the Multicentre Selective Lymphadenectomy Trial‐1 (MSLT‐1) and MSLT‐2 trials, the value of SNB has been well established as a means of accurately staging melanoma and providing regional disease control. However, basing the decision to perform SNB on the primary tumour (T) anatomical stage has significant limitations. Online calculators have improved patient selection for SNB considerably when compared to T stage‐based criteria. In this regard, the MIA risk calculator is recommended in the AJCC 8th edition and performs better than the older MSKCC nomogram [50, 51, 52]. As an example of the tool's effectiveness, take a 33‐year‐old woman with a 0.7 mm superficial spreading, non‐ulcerated melanoma. It is negative for lymphovascular invasion and has 2 mitoses per mm^2^. The AJCC 8th edition estimates such a patient to have a 5%–10% risk of node positivity and that SNB be considered, whereas the MIA calculator estimates this risk to be 16%, making the decision to offer SNB more justifiable.

As with other malignancies, gene expression profile testing has been applied to melanoma prognosis prediction. The MERLIN‐001 prospective trial was presented in 2024 and found that a combined clinicopathological (age and Breslow thickness) and gene expression profile was able to identify melanoma patients with a less than 10% risk of SLN positivity [53]. Other retrospective trials have also shown promise. For instance, when applied retrospectively to 250 clinically node‐negative T1/T2 head and neck melanomas that underwent SNB, the Merlin assay had a 98% negative predictive value with regard to SNB and could potentially have spared 40% of the cohort a SNB [54]. Research is ongoing and to date none of the commercially available tests are funded in Australia or New Zealand [55].

Regarding technique, the dual‐tracer technique with real‐time lymphoscintigraphy is preferred as it allows the nuclear medicine physician to distinguish between 1st order sentinel nodes and 2nd order non‐sentinel nodes [56, 57]. All first‐order lymphoscintigraphy‐mapped lymph nodes and any additional blue node with a direct blue lymphatic draining to it are removed [58].

Occasionally, the surgeon is met with lymphatic drainage to awkward sites such as the deep pelvic or intercostal nodes. Data from the MIA suggests that there is limited benefit in chasing sentinel nodes that may place the patient at higher risk, particularly in inexperienced hands [59]. However, this must be weighed against the pretest probability of a positive SNB, the patient's risk appetite and the likelihood that a positive result would lead to a change in management such as commencement of adjuvant systemic treatment. If the decision is made to leave sentinel nodes in place, it is vital to communicate this to the radiology team to guide imaging surveillance.

Interpreting and using a positive SNB result can be difficult. Prior to the MSLT II and DeCOG‐SLT trials, CLND was generally recommended in these patients. However, these multicentre randomised controlled trials found no improvement in melanoma‐specific survival for patients receiving CLND following a positive sentinel node biopsy and three quarters of patients avoided a lymphadenectomy long term [4, 60]. Consequently, surveillance with or without adjuvant systemic therapy is now standard care.

The degree to which a positive SNB affects a patient's prognosis varies considerably between patients and depends on several factors, including the degree and distribution of nodal disease in the sentinel node and the T stage of the primary [61, 62, 63]. Accurate estimation of an individual's prognosis is important to deciding whether to use adjuvant therapies or not. In particular, adjuvant immunotherapy reduces the relative risk of cutaneous melanoma recurrence by 40%–50% after a positive SNB [8, 63]. However, given that such treatments are expensive, that they come with potential morbidity and that they are subsidised for use only once in a person's lifetime in Australia, the absolute risk reduction (ARR) for the patient is the key question in developing a patient‐centred management plan [8, 62]. ARR refers to the percentage point reduction of a patient's risk. It allows calculation of the number of patients needed to treat in order to prevent one patient recurrence by the formula 1/ARR. For example, for an ARR of 20%, five patients will require treatment to prevent one recurrence [64]. Also noteworthy is that to date there is no proven overall survival benefit to adjuvant therapy for stage 3 melanoma patients. At this time in the evolution of melanoma management SNB remains highly relevant but this might change over time with more data and it may be more selectively applied in the future.

Published in 2024, Stassen et al.'s [62] post‐sentinel node biopsy predictive model is a useful tool to help advise patients regarding their prognosis, which can be extrapolated to estimate their benefit from adjuvant therapies. The model uses six factors to predict recurrence‐free survival, overall survival and melanoma‐specific survival—age, anatomical site of primary melanoma, Breslow thickness, presence of ulceration, sentinel node positivity and the diameter of the largest sentinel node metastasis. A score out of 18 is calculated and this estimates a patient's risk of recurrence and mortality. This can then be used to assess the value of adjuvant systemic therapies to a patient.

It is noteworthy that following publication of the Keynote‐716 and CheckMate‐76K trials of adjuvant pembrolizumab and nivolumab, respectively, for thick melanomas, the relevance of sentinel node biopsy was questioned by some [65, 66]. The trials found significant improvements in EFS when thick melanoma resection was followed by a year of immunotherapy. However, SNB being performed and confirmed negative was an inclusion criterion in both of these trials. Foregoing SNB in clinical stage IIB/C patients would in reality include patients who are pN1a, N2a or N3a, upstaging them to stage IIIC/D. In the absence of this knowledge, the physician and patient do not have a reliable idea of the risk of recurrence and the potential benefit of adjuvant systemic therapy. SNB also significantly improves nodal recurrence rates [67]. Without sentinel node biopsy or immunotherapy, these patients have a nodal recurrence rate of 40%. This risk is lowered to roughly 27% when immunotherapy is administered alone. When SNB is used alone, the nodal recurrence rate is 14%. When combined, the modalities reduce the nodal recurrence rate to 9% [67].

## 4 Macroscopic Stage 3 Lymph Node Disease

Until recently, clinically detected lymph node metastases at presentation (AJCC v8 stage IIIB‐D) portended a poor prognosis [68]. Lymphadenectomy was the mainstay of treatment and distant relapse rates were as high as 70%–80%, implying that regional disease is in most patients a marker of subclinical distant disease [68]. Additionally, surgical morbidity following TLND is significant, particularly for those related to the groin area and is a major concern for patients and clinicians [60, 69].

Targeted therapies that block the BRAF/MEK molecular signalling pathway have improved the outcomes of BRAF‐mutant melanoma patients. Adjuvant trials of combination dabrafenib/trametinib have found significant reductions in melanoma relapse rates with administration of a year of treatment and these drugs remain a key pillar in managing stage 3 and distant metastatic disease [70, 71].

Immunotherapy has further shifted the paradigm for stage IIIB‐D melanoma. Studies have found that local, regional and distant recurrence can be significantly reduced with the administration of 12 months of adjuvant pembrolizumab or nivolumab [9, 68, 72].

Immunotherapy unmasks melanoma cells and allows the patient's immune system to recognise them. When the metastatic melanoma remains in place, more tumour antigens are present to trigger the immune system and the results of neoadjuvant immunotherapy have been startling. This concept was initially validated by the OPACIN proof of concept study and subsequently, by the OPACIN‐neo and PRADO studies amongst others [10, 73, 74, 75, 76, 77].

The SWOG S1801 and NADINA trials have emphatically proven the advantages of administering immunotherapy preoperatively [6, 11]. The SWOGS1801 study saw a relative reduction of 42% in EFS at 2 years follow‐up for patients given three doses of neoadjuvant pembrolizumab and adjuvant pembrolizumab compared to adjuvant pembrolizumab alone. Published last year, the NADINA trial saw further improvements in patient outcomes when combination neoadjuvant ipilimumab and nivolumab were used, with an astounding 83.7% EFS at 12 months follow‐up. Importantly, in the 58% of patients that achieved a major pathological response (MPR—< 10% residual viable melanoma), the immunotherapy was ceased postoperatively, confirming a durable treatment effect. In each of these trials, the duration of neoadjuvant therapy was brief (9 and 6 weeks, respectively) and as such, preoperative progression is uncommon and when present, likely serves to identify patients in whom surgery was otherwise futile.

**FIGURE 1:**
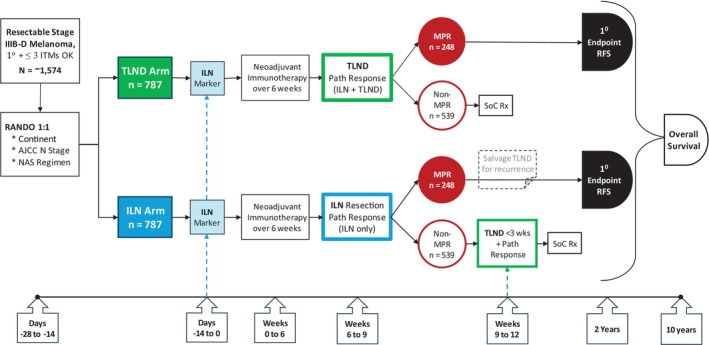
MSLT‐3 trial overview. Resectable stage IIIB‐D melanoma patients will be randomised 1:1 to therapeutic lymph node dissection or selective node dissection after marker placement and combination neoadjuvant IO. Patients treated with selective dissection that have a non‐MPR will undergo TLND. MPR, major pathological response; TLND, therapeutic lymph node dissection.

The efficacy of immunotherapy in treating regional disease has raised the possibility of de‐escalating surgery in the regional nodal basin and sparing patients operative morbidity such as wound complications and lymphoedema. The PRADO trial was a phase 2 response‐driven trial involving the removal of the patient's single dominant involved node after neoadjuvant immunotherapy to assess pathological response [77]. In the 61% of patients who achieved a MPR, CLND was omitted, and no further adjuvant therapy was administered. Patients who did not have a MPR were treated with subsequent TLND with or without adjuvant treatment. The study produced 93% relapse‐free survival and 98% distant metastasis‐free survival rates at 24 months in patients with a MPR. Of 60 patients with a MPR, 59 avoided TLND. Morbidity was significantly reduced in the selective dissection group. A phase 3 study to confirm the safety of de‐escalating the extent of surgery in patients with a MPR after neoadjuvant immune therapy—MSLT3—is soon to begin worldwide recruitment (Figure 1).

## 5 In‐Transit Metastases (ITMs)

In transit metastases affect approximately 6% of melanoma sufferers [78, 79].

When suspected, ITMs should be excised or biopsied with a punch biopsy or core needle biopsy if excision is not possible. A third of ITMs will have associated lymph node metastases and 10% will have distant metastases at the time of diagnosis [78, 79, 80]. As such, regional and distant staging are required with a PET/CT and MRI brain or suitable alternatives.

There is a plethora of management options for ITMs, including surgery, intralesional therapy of various types (depending on availability), systemic therapy, radiotherapy and other regional therapies such as isolated limb infusion/perfusion. There is a lack of data to guide the ideal choice and sequence of these therapies and as such, specialist multidisciplinary discussion is strongly recommended [81, 82, 83]. In most cases, neoadjuvant immunotherapy should be considered. When added to neoadjuvant immunotherapy, oncolytic virus injection (talimogene laherparepvec—T‐VEC) has seen mixed results. However, the MASTERKEY‐265 randomised controlled trial found no difference in PFS or OS when pembrolizumab was combined with T‐VEC or placebo for unresectable disease [84]. It has been contemplated that this was due to the inclusion of stage IV disease and that patients with ITM +/− nodal disease might have benefitted from the addition of T‐VEC; however, this is yet to be proven [83].

In low‐volume, slowly progressive disease, surgery is potentially curative [79, 85]. If resection is decided upon, SNB should be contemplated as a significant proportion of clinically node‐negative patients will return a positive study, particularly if this is likely to be affecting adjuvant treatment decisions [50, 86, 87].

## 6 Oligometastatic Disease

Systemic treatments are the mainstay of management for metastatic melanoma. If drug therapies are unsuitable or unavailable, locoregional treatments are appropriate if agreed to by a multidisciplinary team. Patients with fewer metastases have better outcomes following metastasectomy [88].

For those patients with more widespread disease, definitive systemic treatments are the mainstay. Surgery is not curative and should be reserved for lesions that are symptomatic or pose an imminent threat, such as bulky brain disease or intestinal disease at risk of obstruction. Furthermore, surgery may be used to manage a recalcitrant lesion in the setting of metastatic disease that is oligo‐residual or oligo‐progressive.

## 7 Surveillance

How to follow a patient that has completed their melanoma therapy depends on the risk of recurrence and options for salvage therapy.

Thin and some intermediate thickness melanomas without evidence of metastasis on SNB can be followed clinically and without systemic imaging.

Systemic surveillance with cross‐sectional imaging, such as PET‐CT and MRI brain should be employed in patients with stage 2B disease and above (T3bN0 and greater) (Table 1). Earlier detection of lower volume metastatic disease may convey a better prognosis and provide access to a broader array of therapeutic options. Immunotherapy has given such patients a good chance of long‐term survival.

**TABLE 1:** A guide to imaging surveillance of cutaneous melanoma by stage.

Stage	Imaging modality	Frequency
Stage Ib/IIa, no SNB performed	US of nodal basin	6‐monthly for 3 years Annually years 4–5
Stage IIb/IIc/IIIa, sentinel node metastasis < 1 mm	PET/CT + brain imaging	6‐monthly for 3 years Annually years 4–10
Stage IIIa (> 1 mm deposit)/IIIb/IIIc/IIId/4	PET/CT + MRI brain	3‐monthly for 3 years 6‐monthly years 4–5 Annually years 5–10

Abbreviations: CT, computed tomography; MRI, magnetic resonance imaging; PET, positron emission tomography; US, ultrasound.

## 8 Conclusion

The recent evolution of cutaneous melanoma management with surgical de‐escalation and increasing use of systemic therapy has seen a dramatic improvement in patient outcomes. Therapeutic decision making is becoming more complex, and surgeons must remain current or else risk the welfare of their patients. With the rapid rate of change, discussion of complex cases at a specialised melanoma multidisciplinary meeting or with a colleague who works in that environment is important.

## Disclosure

Matthew Binks: Nil. Alexander C. J. van Akkooi: A/Prof van Akkooi is an Editorial Board member of *ANZ Journal of Surgery* and a co‐author of this article. To minimise bias, they were excluded from all editorial decision‐making related to the acceptance of this article for publication. Honoraria/Consultancy or Advisory Role—Amgen, Bristol‐Myers Squibb, Genmab, Menarini Silicon Biosystems, Merck Serono‐Pfizer, MSD Merck, Neracare, Novartis, Pierre Fabre, Provectus, Sanofi, Sirius Medical, SkylineDx and 4SC. Research Funding—Amgen, Merck Serono—Pfizer, SkylineDx. Alexander M. Menzies: COI: advisory boards BMS, MSD, Novartis, Roche, Pierre‐Fabre and QBiotics. Funding: Alex Menzies is supported by an NHMRC Investigator Grant. Andrew Spillane: Nil.
